# Heterogeneity in regional changes in body composition induced by androgen deprivation therapy in prostate cancer patients: potential impact on bone health—the BLADE study

**DOI:** 10.1007/s40618-023-02150-z

**Published:** 2023-07-17

**Authors:** A. Dalla Volta, C. Palumbo, S. Zamboni, G. Mazziotti, L. Triggiani, M. Zamparini, F. Maffezzoni, L. Rinaudo, M. Bergamini, N. Di Meo, I. Caramella, F. Valcamonico, P. Borghetti, A. Guerini, D. Farina, A. Antonelli, C. Simeone, A. Berruti

**Affiliations:** 1 Medical Oncology Unit, ASST Spedali Civili, Department of Medical and Surgical Specialties, Radiological Sciences and Public Health, University of Brescia, Piazzale Spedali Civili 1, 25123 Brescia, Italy; 2 Division of Urology, Department of Translational Medicine, University of Eastern Piedmont, Maggiore Della Carità Hospital, Novara, Italy; 3 Urology Unit, ASST Spedali Civili, Department of Medical and Surgical Specialties, Radiological Sciences and Public Health, University of Brescia, Brescia, Italy; 4 Department of Biomedical Sciences, Humanitas University, Pieve Emanuele, Italy; 5 Endocrinology, Diabetology and Medical Andrology Unit, Metabolic Bone Diseases and Osteoporosis Section, IRCCS Humanitas Research Hospital, Rozzano, Italy; 6 Radiation Oncology Unit, ASST Spedali Civili, Department of Medical and Surgical Specialties, Radiological Sciences and Public Health, University of Brescia, Brescia, Italy; 7 Endocrinology, Manerbio-Leno, ASST Garda, Montichiari, Italy; 8 Tecnologie Avanzate S.r.l., Turin, Italy; 9 Radiology Unit, ASST Spedali Civili, Department of Medical and Surgical Specialties, Radiological Sciences and Public Health, University of Brescia, Brescia, Italy; 10 Urology Unit, AOUI Verona, Department of Surgery, Dentistry, Pediatrics and Gynecology, University of Verona, Verona, Italy

**Keywords:** Prostate cancer patients, Androgen deprivation, Body composition, Bone health

## Abstract

**Background:**

It is not clear whether changes in body composition induced by androgen deprivation therapy (ADT) in prostate cancer (PC) patients are uniform or vary in the different body districts and whether regional lean body mass (LBM) and fat body mass (FBM) could have an impact on bone health.

**Objective:**

To prospectively evaluate the regional changes in LBM and FBM in PC patients submitted to degarelix; to explore the relationship of regional body composition and bone mineral density (BMD) and bone turnover markers.

**Design, setting, and participants:**

29 consecutive non metastatic PC patients enrolled from 2017 to 2019. FBM, LBM and bone mineral density (BMD) evaluated by dual-energy x-ray absorptiometry (DXA) at baseline and after 12-month of ADT. Alkaline phosphate (ALP) and C-terminal telopeptide of type I collagen (CTX) assessed at baseline, 6 and 12 months.

**Intervention:**

All patients underwent degarelix administration.

**Outcome measurements and statistical analysis:**

*T*-test or sign test and Pearson or Spearman test for continuous variables were used when indicated.

**Results and limitations:**

Median percent increase in FBM ranged from + 14.5% in trunk to + 25.4% in the left leg after degarelix. LBM changes varied from + 2% in the trunk to − 4.9% in the right arm. LBM in both arms and legs and their variations after degarelix directly correlated with ALP and inversely correlated with CTX. Lean mass of limbs, trunk and legs significantly correlated with BMD of the hip, lean mass of the trunk significantly correlated with spine BMD. These are post-hoc analysis of a prospective study and this is the main limitation.

**Conclusions:**

an heterogeneous change in body composition among body district is observed after ADT and bone turnover is influenced by lean mass and its variation. A supervised physical activity is crucial to maintain general physical performance and preserving bone health.

## Introduction

Androgen deprivation therapy (ADT) is the reference treatment in patients with advanced and metastatic prostate cancer (PC) [1]. This treatment does not lead to patient cure but is able to make the disease chronic for a long time [2]. As a consequence patients with advanced PC are often under ADT for several years and are exposed to the long-term side effects of this treatment. Androgens are important determinants of Lean Body Mass (LBM) and Fat Body Mass (FBM) in men [3]. Prolonged androgen deprivation from ADT could place PC patients at risk of sarcopenic obesity by LBM reduction and FBM increase [4, 5] and on an accelerated trajectory to disability. Sarcopenic obesity is in fact notoriously associated with an increased risk of diabetes, metabolic syndrome and cardiovascular mortality [6].

Moreover, in relation to the increasing observations of an interaction between fat mass, lean mass and skeletal fragility in PC patients under ADT [7, 8], sarcopenic obesity could also favour bone fragility fractures, due to mechanical and biochemical cross-talking between muscle and bone [9] and an increased risk of falls.

ADT has been based for decades on the administration of luteinizing hormone releasing hormone analogs (LHRH-As)[1] to which LHRH antagonists, such as degarelix, have recently been added [10]. Dual-energy x-ray absorptiometry (DXA), which is commonly used to measure bone mineral density (BMD), represents also a reference method for the assessment of human body composition in the research field [11]. Many studies have used the DXA scan to evaluate changes in body composition after ADT in patients with prostate cancer [4] and the same method has frequently been used to assess the impact of supervised physical activity on fat and lean mass in this patient category [12]. In these studies, only changes in total LBM and FBM were taken into consideration.

We recently conducted the BLADE study (Bone mineraL mAss Dexa dEgarelix), a phase IV study designed to obtain explorative information on DXA measurement changes in LBM and FBM in patients with non-metastatic PC treated with degarelix. Data on changes in total LBM and FBM, recently published [13], showed that degarelix administration was associated with an increase in FBM while LBM was substantially unchanged. However, these data do not clarify if the changes in body composition were uniform or if there was a difference in relation to the different body districts. Indeed, there is consistent evidence that distribution of LBM and FBM could have impact on skeletal mass and strength at different skeletal sites [14–17]. Since the DXA scan is able to evaluate both the whole body and regional lean mass and fat mass [18], the purpose of this paper was to evaluate whether the changes in LBM and FBM in the patients included in the BLADE study are uniform or there is a regional difference. As secondary endpoint we also explored whether an heterogeneity in changes in LBM may have an impact on bone health.

## Patients and methods

### Trial design and endpoints

BLADE is a single-center, prospective, interventional phase IV study (clinicalTrials.gov NCT03202381, EudraCT Number 2016-004210-10) conducted at the Prostate Cancer Unit of the Azienda Socio Sanitaria Territoriale degli Spedali Civili and Università degli Studi of Brescia. The study was carried out in accordance with the Declaration of Helsinki Principles and Good Clinical Practices and was approved by the Ethics Committee of Brescia (approval number NP2540). All patients provided a written informed consent. Male patients with histologically confirmed prostate cancer without bone metastasis at bone scintigraphy, judged eligible to ADT according to current guidelines recommendations [19, 20] after a multidisciplinary discussion, were enrolled. Eligibility criteria have been published elsewhere [13]. Degarelix was administered as a subcutaneous injection with a starting dose of 240 mg, followed by a maintenance dose of 80 mg every 28 days. After 12 months, treatment with degarelix was continued as clinically indicated.

### Assessment of regional lean body mass and fat body mass by dual x-ray absorptiometry

DXA measurements for assessing bone mineral density and body composition parameters were performed at baseline and 12 months, using Hologic QDR-4500W instrumentation (Hologic Corporation, Waltham, Massachusetts). Data were analysed by a dedicated Endocrinologist (FM).

DXA measurements related to whole body DXA scans were extracted from Apex Software version 3.4.

The densitometric image of each patient was divided, following the manufacturer's instructions, into different body districts including arms, legs, trunk, head and other derived regions such as the android and gynoid zone.

BMD, BMC, fat free mass and fat mass were assessed for every region of interest, where fat free mass was provided by the software in terms of lean soft tissue plus bone mineral content (BMC). Despite the lean mass measured by DEXA counts also skin, connective tissue and some lean components within the adipose tissue [21], it still correlates highly with TC and MRI measurements and represents a good approximation of the real muscle mass [22].

Other DXA derived body composition parameters, such as fat mass index (FM/ H2) (FMI), appendicular lean mass index (ALM/H2) (ALMI) and Trunk/Appendicular ratio were then calculated to complete the analysis and the patient characterization.

### Biomarkers

Blood chemistry and bone turnover markers: alkaline phosphate (ALP) and C-terminal telopeptide of type I collagen (CTX) were assessed at baseline, 6 and 12 months. CTX serum levels were measured using the ElectroChemiLuminescenceAssay (ECLIA) kit Elecsys beta-CrossLaps/serum (Roche Diagnostic, Germany) using Cobas e411instruments (Roche); normal ranges were < 0.704 ng/ml (men of the age between 50 and 70), < 0.854 ng/ml (men > 70) with a repeatability CV% of 2.6. Bone-ALP serum levels were determined in a twostep procedure. Briefly, total ALP serum activity was measured using the colorimetric method ALP2 (Roche) using Cobas c701 instruments (Roche); normal ranges were 50–116 U/L ± 0.6 with a repeatability CV% of 0.7. Samples were then subjected to electrophoretic separation to separate the different ALP isoforms using the G26 automated system (Sebia, France) equipped with the Interlab specific kit (Italy). Bone-ALP activity was calculated as fraction of the total ALP activity related to the percentage of densitometric analysis of the electrophoretic migration.

### Statistical analysis

The normal distribution of continuous variables was checked by looking at distribution plots and tested with the Shapiro–Wilk test. Distributions of the following parameters have not been approximated to a normal: TOT Fat (g), Trunk Fat (g), Android%Fat, VAT mass (g), VAT Volume (cm^3^), VAT area (cm^2^). Variables with normal distributions were represented by mean ± standard deviation, while non-normal variables were presented by median and interquartile range. Differences between parameters at baseline and 12-months were computed as percentage changes, and to test if these changes were significantly different from 0, we used one sample t-test, or alternatively the non-parametric sign test.

Variations of CTX, ALP, LBM, FBM, and BMD were considered as percentage and correlations between variables either at baseline and 12-months, as well as correlations between variable changes were expressed as Spearman R.

Statistics were performed using R and SPSS (IBM Corp. Released 2015. IBM SPSS Statistics for Windows, Version 23.0. Armonk, NY: IBM Corp.)

## Results

**Fig. 1:**
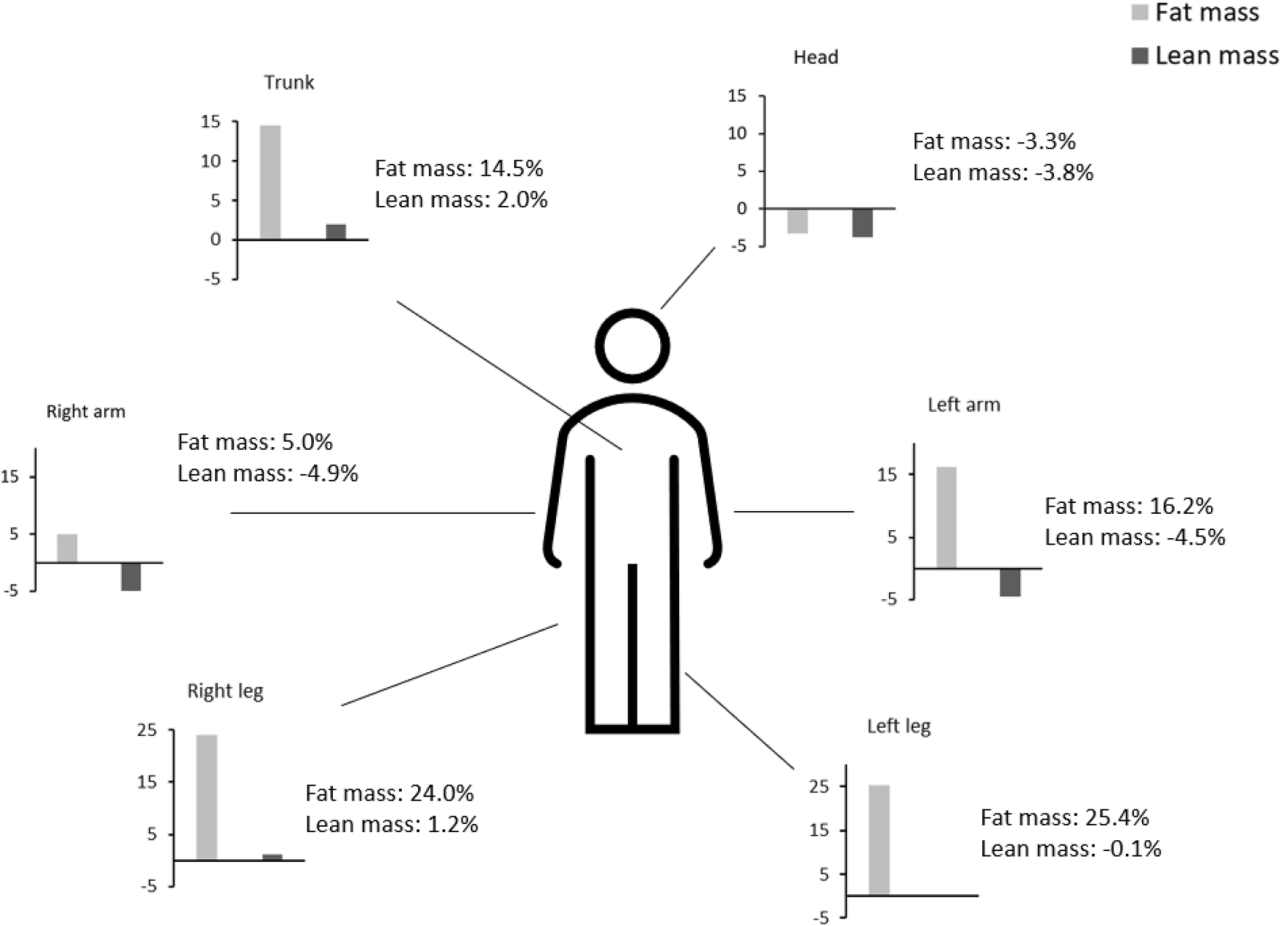
Percent changes in fat and lean mass in different body districts during 12-month Degarelix treatment

Twenty-nine patients were included in the BLADE study and their characteristics have been described elsewhere [8]. As shown in Table 1, a significant increase in FBM was observed in left arm, right and left legs, trunk (Table 1) with a relevant heterogeneity. FBM in right arm did not change, while it even decreased in head district. The heterogeneity of the change in FBM according to the body districts is clearly highlighted in Fig. 1, which describes the distribution of the averages of the percentage variations ranging from a median percent increase of 14.5% in trunk to 25.4% in the left leg. The % android fat increased by 8.3%, while the % gynoid fat increased by 19.2%, leading to a decrease in android/gynoid ratio of 8.9%. Conversely visceral adipose tissue did not vary significantly in terms of either mass (grams), volume (cm^3^) and area (cm^2^) (Table 1).

**Table 1:** Changes in fat body mass densitometric parameters during 12-months Degarelix treatment

	Before Degarelix	After Degarelix	Percent variation	p
BMI	26.8 ± 4.3	27.7 ± 4.2	3.7 ± 4.8	** < .001**
TOT Fat (g)*	18,957.4 (15,888.2; 26,588.8)	23,002.9 (17,823.0; 27,649.3)	10.0 (5.5; 22.3)	** < .001**
Left Arm Fat (g)	1236.4 ± 500.3	1393.2 ± 495.8	16.2 ± 22.1	**.005**
Right Arm Fat (g)	1335.6 ± 529.3	1362.1 ± 509.2	5.0 ± 21.9	.623
Left Leg Fat (g)	2843.2 ± 1243.5	3450.5 ± 1327.8	25.4 ± 20.4	** < .001**
Right Leg Fat (g)	3028.9 ± 1327.5	3612.8 ± 1305.1	24.0 ± 20.5	** < .001**
Trunk Fat (g)*	10,869.6 (8432.3–14,972.9)	12,459.8 (8834.2—15,393.9)	8.0 (2.5—20.9)	** < .001**
Head Fat (g)	1083.6 ± 127.3	1045.3 ± 129.2	-3.3 ± 8.3	**.036**
Android %Fat*	32.5 (26.5–37.8)	34.3 (29.2—40.1)	6.6 (-5.2—13.0)	**.012**
Gynoid %Fat	25.5 ± 5.6	29.9 ± 4.9	19.2 ± 13.5	** < .001**
Android/Gynoid Ratio	1.3 ± .2	1.1 ± .2	− 8.9 ± 10.0	** < .001**
Trunk/Appendicular Fat ratio	1.4 ± .3	1.3 ± .3	− 3.9 ± 14.7	.114
VAT mass (g)*	817.0 (697.0–1025.0)	809.5 (668.3–1015.8)	3.2 (− 2.6–19.1)	.125
VAT Volume (cm^3^)*	883.0 (753.0–1108.0)	875.5 (722.8–1098.3)	3.1 (− 2.6–19.1)	.125
VAT area (cm^2^)*	169.0 (145.0–213.0)	167.5 (138.5–210.5)	3.4 (− 2.6–18.6)	.130
FMI*	6.6 (5.2—8.9)	7.5 (6.0–9.6)	10.0 (5.5–22.3)	** < .001**

Normally distributed variables are presented as mean and SD, not normally distributed variables(*) are presented as median and interquartile range (IQR) and comparisons were performed by one sample t-test on percent variation (test val. = 0) or sign test, for normally or non-normally distributed variables, respectively

*BMI* body mass index, *TOT* total, *VAT* visceral adipose tissue, *FMI* fat mass index

As regard as LBM, it did not significantly vary in trunk and both legs, but consistently decreased in head and both left and right arms (Table 2). The heterogeneity in % changes in LBM according to body districts is described in Fig. 1. The average percent change in LBM varied from + 2% in the trunk to –4.9% in the right arm.

**Table 2:** Changes in lean body mass densitometric parameters during 12-months Degarelix treatment

	Before Degarelix	After Degarelix	Percent variation	*p*
Left Arm Lean (g)	3244.0 ± 618.5	3066.2 ± 464.0	-4.5 ± 10.2	**.009**
Right Arm Lean (g)	3359.5 ± 649.2	3190.3 ± 730.3	-4.9 ± 10.5	**.030**
Left Leg Lean (g)	8812.0 ± 1402.6	8768.5 ± 1286.7	-.1 ± 6.7	.721
Right Leg Lean (g)	8882.4 ± 1391.9	8956.5 ± 1367.1	1.2 ± 7.8	.612
Trunk Lean (g)	29,176.6 ± 4533.7	29,626.5 ± 4306.7	2.0 ± 7.7	.272
Head Lean (g)	3686.7 ± 382.1	3538.2 ± 380.1	-3.8 ± 7.0	**.006**
TOT Lean (g)	57,161.2 ± 8340.0	57,146.2 ± 7660.9	.3 ± 5.7	.981
Lean/Ht^2^	19.0 ± 2.3	19.0 ± 2.0	.3 ± 5.7	.995
ALMI (Appendicular Lean/Ht^2^)	8.1 ± 1.1	8.0 ± .9	-.9 ± 5.9	.286

Normally distributed variables are presented as mean and SD, and comparisons were performed by one sample t-test on percent variation (test val. = 0) *TOT* total; *Ht*^*2*^ height squared, *ALMI* appendicular lean mass index

**Fig. 2:**
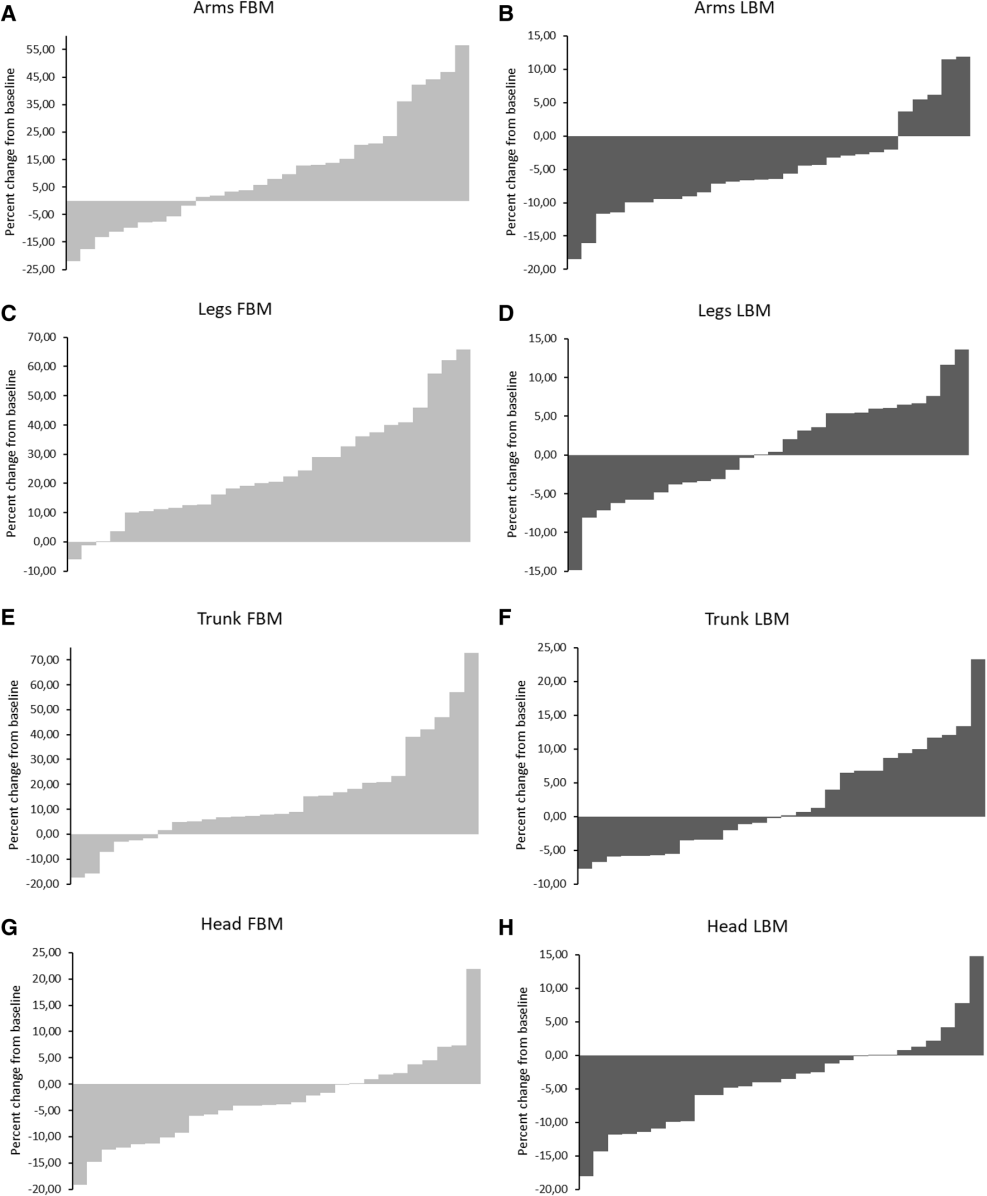
Waterfall plots for regional changes in fat body mass (FBM) and lean body mass (LBM) from study baseline to month 12 in different body districts: arms (**A**, **B**), legs (**C**, **D**), trunk (**E**, **F**), head (**G**, **H**). Histograms represent the percent change from baseline for each patient

Waterfall plots for % changes in each patients of FBM and LBM in the different body districts (head, arms, trunk and legs) from baseline to month 12 of degarelix administration are depicted in Fig. 2.

### Relationship between regional lean mass and bone health

Since in the BLADE study appendicular lean mass index (ALMI), which is the sum of lean mass in legs and arms, was inversely associated with bone fragility [8], we separately explored the relationship between lean mass in legs and arms with markers of bone turnover at baseline conditions as well as the relationship between their changes after degarelix administration. As shown in Table 3a, at baseline conditions an inverse relationship was found between serum CTX levels and either lean mass in legs (*r* = − 0.32, *p* = 0.09) or arms (*r* = − 0.50, *p* = 0.007) (Table 3a), the last relationship attaining the statistical significance.

**Table 3:** **a** Baseline relationships between CTX and ALP and Legs and Arms Lean Body Mass. **b** Relationships between percent variation of lean mass in arms and legs and percent variation of CTX and ALP

a		
	CTX	ALP
Arm Lean (g)	− .498*	.268
Leg Lean (g)	− .321	.308

b		
	CTX	ALP
Arm Lean (g)	− .146	.518*
Leg Lean (g)	− .476*	.333

Data are Spearman R

*Significant relationships

Conversely, a direct relationship was found between ALP and LBM both in legs (*r* = 0.27, *p* = 0.17) and arms (*r* = 0.31, *p* = 0.11), although without attaining the statistical significance (Table 3a). As regards as the changes after degarelix exposure, as shown in Table 3b, a significant inverse relationship was found between variation in lean mass of both legs and changes in CTX serum levels (*r *= − 0.48, *p* = 0.010), while this relationship was not statistically significant with lean mass in arms (*r* = − 0.15, *p* = 0.46) (Table 3b). A direct relationship was found between changes in lean mass either in arms (*r* = 0.52, *p* = 0.005) or in legs (*r* = 0.33, *p* = 0.08) and ALP (Table 3b), the changes in the arms being statistically significant.

We also evaluated exploratively the correlations between bone turnover marker levels (CTX and ALP) and either lean body mass at trunk or fat mass at trunk, arms and legs at baseline conditions and no significant relationships were found except for trunk lean mass and ALP (*r* = 0.44, *p* = 0.018). Similarly non correlations were found between changes in bone turnover markers and changes in lean mass at trunk and fat mass at trunk, legs and arms, respectively (data not shown).

Finally, we analyzed the correlations between the lean mass of the various districts with the BMD of the spine and femur. The results showed highly significant correlations between the lean mass of the limbs, trunk and legs with the BMD of the hip, while only the lean mass of the trunk was significantly correlated with the BMD of the spine (Table 4).

**Table 4:** Relationships between regional lean mass and BMD at spine (L2-L4) and hip at baseline (**A**) and correlation of % changes from baseline after 12-months Degarelix treatment (**B**)

**a**		
	BMD L2-L4	BMD LEFT HIP
Arm Lean (g)	.106	.543*
Leg Lean (g)	.256	.688*
Trunk Lean (g)	.449 *	.754*
Arm Fat (g)	.181	.449*
Leg Fat (g)	.175	.507*
Trunk Fat (g)	.140	.551*

b		
	BMD L2-L4 (% changes)	BMD LEFT HIP (% changes)
Arm Lean (% changes)	.107	− .172
Leg Lean (% changes)	− .154	.098
Trunk Lean (% changes)	.163	− .221
Arm Fat (% changes)	− .301	.181
Leg Fat (% changes)	− .231	.212
Trunk Fat (% changes)	− .271	.127

Data are Spearman R

*BMD* bone mineral density

*Significant relationships

## Discussion

The BLADE study was designed to evaluate changes in body composition, bone mineral density assessed with the DEXA scan and changes in bone turnover markers before and after administration of degarelix. The results, published elsewhere, showed, as expected, that degarelix administration was associated with an increase in FBM, a reduction in BMD and an increase in turnover markers [8, 13]. However there were no changes in lean mass and ALMI [13], contrary to what was observed after the administration of LHRH agonists [4, 7]. DXA is able to obtain precise information on district changes in body composition [23]. The present study showed that the increase in FBM showed considerable variations according to the various body districts and, within the same body district, in relation to the body hemisome. The increase in FBM was relevant at the trunk and legs, with a characteristic gynoid distribution.

As for the lean body mass, the results showed that only the LBM of the legs and trunk did not change, while the LBM of the upper limbs and head showed a notable variation.

Noteworthy, in the previous paper we reported that total LBM did not change after degarelix, in this paper we showed that the non-change in LBM appears to be confined to the legs and trunk but not to the arms and other body districts.

The uneven variation of FBM after degarelix with substantial increase in the lower limbs and trunk is attributable to the effect of androgen deprivation which induces a gynoid distribution of body fat. Conversely, the notable difference in the variations of the lean mass between the lower and upper limbs reflects the different demands on the muscles of these districts, the legs are continuously solicited by walking while the physical activity of the upper limbs in the elderly subject is usually lower and this would explain the greater loss of muscle mass in these body districts. These data are interesting and deserve confirmation.

In recent years, numerous studies have demonstrated the importance of supervised exercise training in preserving muscle mass in patients undergoing androgen deprivation therapies [12]. The results of the present study may be of help in orienting physical exercise towards those muscles with the highest risk of decrease after hormonal treatment.

A growing series of studies have shown an important role of lean mass in maintaining bone health [24–26]. The condition of sarcopenia negatively interact with bone not only mechanically and by increasing the risk of fall, but also in reason of a biochemical cross-talking between muscle and bone [27].

In our subjects treated with degarelix, differently from other clinical settings [14, 28–30], regional changes in FBM did not have direct effects on parameters of skeletal health, whereas the impact of regional changes in LBM on bone turnover and mass resulted to be more important.

Published data from the BLADE study showed a strong inverse relationship between ALMI and CTX at baseline, which is a marker of osteoclastic activity. A similar inverse correlation was found between changes in ALMI after degarelix and changes in CTX. On the other hand, direct correlations between basal ALMI and its variations and basal ALP (a bone formation marker) and its variations after degarelix were observed [8]. These opposite correlations suggest that the loss of lean mass after androgen deprivation can favour uncoupled bone resorption and formation leading to rapid alterations of bone quality, as in other forms of secondary osteoporosis.

In this study we explored the effect of regional LBM and bone turnover. Regarding the turnover markers, CTX and ALP showed an inverse and direct correlation with LBM in both arms and legs, respectively. A similar relationship was observed between changes in LBM in both arms and legs and changes in CTX and ALP after degarelix. These data suggest that the arm muscles, although much smaller than that of the legs, play a role in supporting bone strength and that the prevention of bone health degradation after androgen deprivation therapy involves strengthening the musculature of both limbs.

However, the circulating markers are expression of total bone turnover but do not provide information on the district effect of the musculature on the skeleton. For this reason, we evaluated the correlations between regional lean mass and BMD of the spine and femur. The results showed a strong correlation between lean mass of legs, arms and trunk and hip BMD at baseline conditions. However, only trunk lean mass was shown to correlate with spine BMD. This finding is consistent with previous observations reporting an association between size of psoas muscle and BMD at lumbar spine[31]. As a matter of fact, our study suggests that DXA measurement of regional distribution of LBM might be as reliable as other more invasive diagnostic tools in identifying subjects with sarcopenia at risk of spine fractures.

In addition these data suggest the importance of supporting the trunk muscles to contain the increase in bone fragility in the vertebral column.

In conclusion, this study shows for the first time a large degree of heterogeneity in the changes in body composition among body district and underline the strict relationship between lean mass and bone health.

These results support the implementation of supervised physical activity not only to maintain general physical performance and prevent the risk of falling, but also to preserve bone health as much as possible. The prospective design and the strong correlations observed despite the low number of patients enrolled are the strengths of this study. This study suffers from several limitations, the analyses presented in this paper were not planned and are therefore post hoc; the major parameters to evaluate sarcopenia, such as indexes belonging to muscle performance (i.e. grip test and others) [6], were not assessed; and the number of falls in the previous years before entering the study, as an indirect sign of sarcopenia, were not collected.

Future studies designed to prospectively test the interaction between total and district lean mass and bone health are warranted. In these studies, a better characterization of sarcopenia including muscle performance tests will need to be performed.

## Patient summary

- Androgen deprivation in prostate cancer patients induces changes in body composition which vary considerably in different body districts.
- Muscle mass decrease in legs and arms negatively impacts on bone health.
- A supervised physical activity is needed to maintain a general physical performance and preserve bone health in prostate cancer patients under androgen deprivation.
